# A tetramerization domain in prokaryotic and eukaryotic transcription regulators homologous to p53

**DOI:** 10.1107/S2059798323001298

**Published:** 2023-03-01

**Authors:** Nerea Bernardo, Isidro Crespo, Anna Cuppari, Wilfried J. J. Meijer, D. Roeland Boer

**Affiliations:** a Experiments Division, ALBA Synchrotron Light Source, Carrer de la Llum 2–26, 08290 Cerdanyola del Vallès, Catalunya, Spain; b Centro de Biología Molecular ‘Severo Ochoa’ (CSIC–UAM), Universidad Autónoma de Madrid, Calle Nicolás Cabrera 1, Canto Blanco, 28049 Madrid, Spain; University of Queensland, Australia

**Keywords:** transcription regulation, DNA looping, tumor suppression, structural biology

## Abstract

Transcriptional regulation usually requires the action of several proteins that either repress or activate a promotor of an open reading frame. These proteins can counteract each other, thus allowing tight regulation of the transcription of the corresponding genes, where tight repression is often linked to DNA looping or cross-linking. Here, the tetramerization domain of the bacterial gene repressor Rco from *Bacillus subtilis* plasmid pLS20 (Rco_pLS20_) has been identified and its structure is shown to share high similarity to the tetramerization domain of the well known p53 family of human tumor suppressors, despite lacking clear sequence homology. In Rco_pLS20_, this tetramerization domain is responsible for inducing DNA looping, a process that involves multiple tetramers. In accordance, it is shown that Rco_pLS20_ can form octamers. This domain was named TetD_loop_ and its occurrence was identified in other *Bacillus* species. The TetD_loop_ fold was also found in the structure of a transcriptional repressor from *Salmonella* phage SPC32H. It is proposed that the TetD_loop_ fold has evolved through divergent evolution and that the TetD_loop_ originates from a common ancestor predating the occurrence of multicellular life.

## Abstract 2

The tetramerization domain of the bacterial gene repressor Rco from *Bacillus subtilis* plasmid pLS20 was identified. The structure shows high similarity to the tetramerization domain of the p53 family of human tumor suppressors, despite having low sequence homology.

## 1. Introduction

Proper gene regulation is essential for every organism to adjust the expression profile of its encoded genes to changing environmental conditions. The mechanisms of transcriptional regulation are very different in prokaryotes and eukaryotes, and are generally more complex in the latter. For example, in eukaryotes the genomes are packed in more sophisticated ways and transcriptional regulators rely less on sequence specificity for DNA response elements (REs; Youssef *et al.*, 2019 ▸). The reason for this higher level of sophistication is most likely related with intercellular communication in multicellular organisms, which becomes clear when considering situations in which gene regulation is disturbed and causes disease (Lee & Young, 2013 ▸). The well studied human tumor suppressor protein p53 illustrates the importance of proper transcriptional regulation (Vogelstein *et al.*, 2000 ▸; Lane & Levine, 2010 ▸). Mutations in this protein have been associated with the occurrence of tumorigenesis for over four decades (Rivlin *et al.*, 2011 ▸; Perri *et al.*, 2016 ▸).

The active control of the conformation of the DNA duplex regulates access to promoter regions and thereby makes an important contribution to gene regulation. An example of this is long-range dsDNA looping, which is not to be confused with the formation of short hairpin loops in ssDNA. In long-range looping, two DNA elements are brought into close proximity by introducing a kink or strong bend in the DNA located between the two elements. DNA looping occurs frequently in both prokaryotes as well as eukaryotes (Cournac & Plumbridge, 2013 ▸; Vilar & Saiz, 2005 ▸; Morelli *et al.*, 2009 ▸). Examples of transcriptional regulation through DNA looping in prokaryotes are found in the metabolic genes *ara*, *gal*, *lac* and *deo* and in phage systems (for a review, see Matthews, 1992 ▸). In addition, the human tumor suppressor protein p53, which represents a large family of homologs in metazoa, induces the DNA bending required for binding (McKinney & Prives, 2002 ▸). Interestingly, all of these regulators form homotetramers and have been shown to be essential for DNA looping, even though the folds of the proteins involved do not share any structural similarity. This suggests that the tetrameric quaternary structure provides a particular functional advantage in the formation of DNA loops, which may be related to the cooperativity in the stability conferred by having two DNA-recognition anchoring points at both extremes of the loop region.

p53 has been studied extensively and many functions have been attributed to this protein in processes related to, amongst others, human development and DNA repair and metabolism (Gaglia *et al.*, 2013 ▸; Lane & Levine, 2010 ▸). It consists of five domains: two transcription-activation domains (TADs; residues 1–40 and 40–61), a proline-rich region (residues 64–92), a DNA-binding domain (DBD; residues 98–303), a nuclear localization signal-containing region (residues 303–323), an oligomerization domain (residues 323–365) and a C-terminal basic domain (residues 363– 393). The TADs are important for the transactivation of different target genes. The DBD recognizes the p53 recognition element (p53RE), which consists of two copies of a 5′-RRRCWWGYYY-3′ sequence (IUPAC nomenclature, where R is A or G, Y is C or T and W is A or T) separated by a spacer of 0–13 bp (Brázda & Coufal, 2017 ▸; El-Deiry *et al.*, 1992 ▸). The oligomerization domain induces tetramerization and possibly other oligomerization states of p53, which has been amply documented (reviewed in Chène, 2001 ▸). We will refer to this domain as p53Tet. The C-terminal domain is highly charged due to a high lysine content and is intrinsically disordered. This domain has a dual function: the positive charge enhances the affinity for DNA and at the same time is responsible for recruiting other factors. p53 is also able to form DNA loops and link DNA across large distances due to the combined action of the DBD and the p53Tet domain, which itself does not bind DNA but rather links DBD-bound DNAs (Stenger *et al.*, 1994 ▸; Kearns *et al.*, 2016 ▸; Brázda & Coufal, 2017 ▸). Many of the oncogenic mutations occur in the DBD (Perri *et al.*, 2016 ▸), but several have been mapped to the tetramerization domain (reviewed in Chène, 2001 ▸; see references in Kamada *et al.*, 2011 ▸; Petitjean *et al.*, 2007 ▸). In addition, mutations in p53Tet cause Li–Fraumeni syndrome, a hereditary disease that conveys a high disposition to develop early-onset neoplasms (Etzold *et al.*, 2015 ▸).

Here, we report a structural analog of the oligomerization domain of p53 from the conjugative plasmid pLS20 of the Gram-positive (G+) bacterium *Bacillus subtilis*. This transcriptional regulator, named Rco (Singh *et al.*, 2013 ▸), forms tetramers in solution (Ramachandran *et al.*, 2014 ▸; Crespo *et al.*, 2020 ▸; Singh *et al.*, 2013 ▸) and represses the main conjugation promoter P_c_ that controls the transcription of genes 28–74. The promotor contains 11 copies of the sequence 5′-CAGTGAAA-3′ and variations thereof (Ramachandran *et al.*, 2014 ▸), which are likely to be the recognition sites for Rco_pLS20_. Rco_pLS20_ controls its own expression by regulating the activity of the overlapping and divergently oriented P_r_ promoter (reviewed in Meijer *et al.*, 2021 ▸). The simultaneous regulation of the P_c_ and P_r_ promoters involves Rco_pLS20_-mediated DNA looping, which is achieved through the binding of Rco_pLS20_ to two operators separated by 75 bp and located near the P_c_ and P_r_ promoters, each containing multiple direct repeats of the Rco-recognition element (rcoRE; Ramachandran *et al.*, 2014 ▸). One of the operators, O_II_, overlaps with the P_c_ and P_r_ promoters and contains at least six direct repeats of the rcoRE. The other operator, O_I_, contains at least four direct repeats of the rcoRE, which are convergently oriented with respect to the rcoREs in operator O_II_. DNA recognition by Rco is most likely to be achieved through a conserved helix–turn–helix (HTH) domain at its N-terminus, which is followed by a sequence of hitherto unknown function at its C-terminus. Given the presence of at least ten rcoREs in the promotor region to which Rco binds, it can be expected that at least two Rco tetramers bind to this region.

We have previously solved the structure of Rap_pLS20_ (Crespo *et al.*, 2020 ▸), which is the response regulator that binds Rco_pLS20_, thereby activating the conjugation promotor and allowing expression of the conjugation operon (Singh *et al.*, 2013 ▸). The aim of this study was to understand the structural mechanism of transcriptional regulation of Rco_pLS20_. For this purpose, we further analyzed the oligomerization behavior of Rco_pLS20_ and identified the oligomerization domain. We confirm the formation of tetramers and show that Rco_pLS20_ can also form octamers under specific conditions. Furthermore, we present the crystal structure of the tetramerization domain of Rco_pLS20_. The domain encompasses 35 residues of the C-terminal region and is structurally homologous to the tetramerization domain of the human oncogene p53. As this fold is implicated in the formation of DNA loops, we designate domains that have this fold TetD_loop_. We define a motif for TetD_loop_ and suggest that the TetD_loop_ domain is ubiquitous among all kingdoms of life.

The implications of the occurrence of TetD_loop_ in prokaryotes and eukaryotes are profound. First of all, the structures suggest that the fold precedes the appearance of multicellular life, which had not been considered (see, for example, Joerger *et al.*, 2014 ▸). This implies that these proteins share a common ancestor and hence are ubiquitous. Secondly, it suggests that a basic paradigm of gene regulation through DNA looping exists among prokaryotes and eukaryotes, and we believe that the possible parallels between the mechanism of action of p53 and Rco_pLS20_ should be further investigated.

## 2. Materials and methods

### 2.1. Production and purification of Rco_pLS20_

Cloning, expression, isolation and purification of Rco_pLS20_ were performed as described previously (Crespo *et al.*, 2020 ▸). Typically, a yield of 20 mg Rco_pLS20_ was obtained from 10 g cell pellet. Purity was assessed to be >95% by SDS–PAGE followed by Coomassie Blue staining. Protein concentration was determined from the absorbance at 280 nm on a Nanodrop 2000 spectrophotometer (ThermoFisher Scientific) using an extinction coefficient ɛ(1%) of 2.93. Protein was used for assays immediately where possible or stored in aliquots at −80°C.

### 2.2. Crystallization of RcoTet_pLS20_

Rco_pLS20_ was concentrated to 17 mg ml^−1^ using an Amicon Ultra 15 ml centrifugal filter (Merck Millipore) with a cutoff of 10 kDa in a buffer consisting of 250 m*M* NaCl, 20 m*M* Tris pH 8.0. Concentrated Rco_pLS20_ was gently mixed with a previously annealed double-stranded oligonucleotide (Biomers.net, Germany) with forward sequence 5′-GTCAGTGAAAAA-3′ in a 2:1 (protein:DNA) stoichiometry. The crystals giving the highest resolution data were obtained by the sitting-drop vapor-diffusion method at 18°C by equilibration of drops consisting of 100 nl protein solution and 100 nl crystallization buffer [0.1 *M* HEPES pH 7.5, 28%(*v*/*v*) PEG 600] against 100 µl crystallization buffer in the reservoir. The crystals took three months to grow and were harvested for X-ray diffraction data collection by cryocooling them by direct transfer from the crystallization drop into liquid nitrogen.

### 2.3. Data collection and processing

Data collection was performed on the BL13-XALOC beamline at the ALBA Synchrotron Light Source at 100 K. Data were processed with *AutoPROC* (Vonrhein *et al.*, 2011 ▸) using anisotropic resolution cutoffs (see Table 1 ▸). The structure was determined *de novo* using *ARCIMBOLDO* (Rodríguez *et al.*, 2009 ▸) followed by automated model building in *Phenix* version 1.12-2829 (Adams *et al.*, 2010 ▸). The structure was refined using *Phenix* interspersed with manual adjustments in *Coot* version 0.8.9.2205 (Emsley *et al.*, 2010 ▸). The refinement statistics are given in Table 1 ▸. The structure was deposited in the PDB with accession code 8bny.

### 2.4. Calculation of an *AlphaFold* model of full-length Rco_pLS20_

*AlphaFold* version 2.1.0 (Jumper *et al.*, 2021 ▸) was used to generate five models of full-length Rco_pLS20_ (UniProt entry E9RIY8). The five models are essentially the same and the highest ranking model was used for analysis.

### 2.5. Database searches and figures

*BlastP* (Altschul *et al.*, 1990 ▸) searches were performed using residues Ser124–Asp161 of the Rco_pLS20_ sequence. Figures were prepared using *PyMOL* version 2.3 (Schrödinger). Superpositions were performed using the built-in ‘align’ function in *PyMOL*. *PDBeFold* from EMBL–EBI was used to identify folds similar to that of Rco_pLS20_ in the PDB (Krissinel & Henrick, 2005 ▸).

### 2.6. Size-exclusion chromatography (SEC) assays

25 µg Rco_pLS20_ (25 µl) were injected into a Superdex 200 Increase 5/150 GL column (GE Healthcare) that had been equilibrated with buffers at different pH values. For pH 5, the column was equilibrated with 500 m*M* NaCl, 20 m*M* citrate buffer pH 5. For pH 8, 500 m*M* NaCl, 20 m*M* Tris pH 8 was used. For pH 10, 500 m*M* NaCl, 20 m*M* glycine–NaOH pH 10 was used. Elution was performed at a flow rate of 0.2 ml min^−1^. The elution was continuously monitored at wavelengths of 280 and 260 nm. Estimation of the molecular weight (MW) was performed from the elution volume *V*_el_ of the detected peaks using an in-house calibration of the relation between log(MW) and *V*_el_ of proteins with known MW. The equation derived from this calibration was *V*_el_ = −0.6815log(MW) + 5.1906, with *R* ^2^ = 0.933.

## 3. Results

### 3.1. Crystal structure of the tetramerization domain of Rco_pLS20_

**Figure 1:**
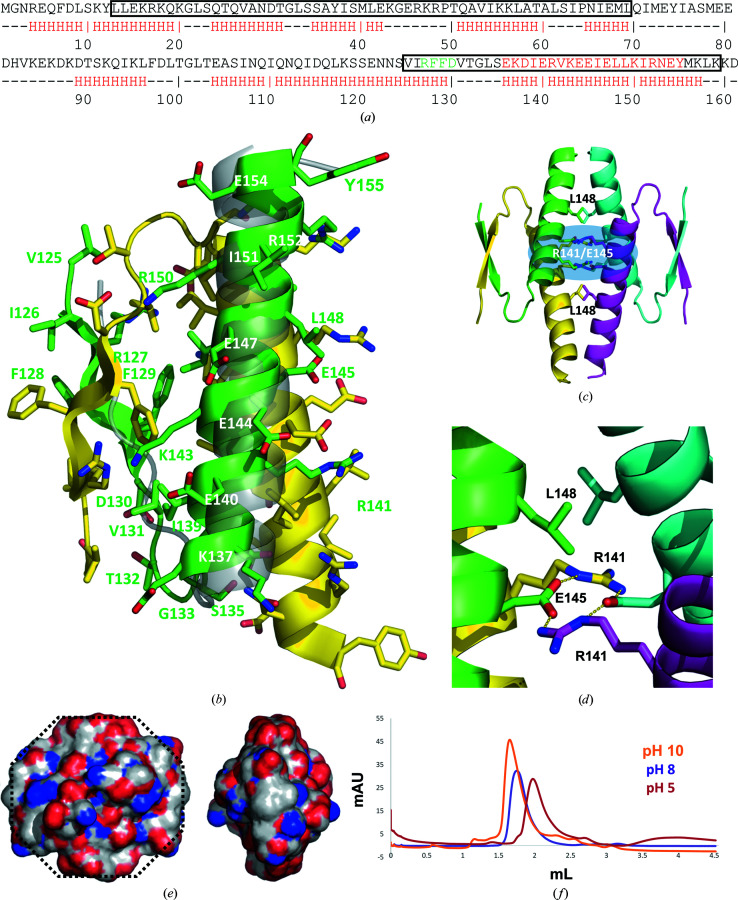
Overview of the structure and oligomerization behavior of RcoTet_pLS20_. (*a*) The sequence and secondary-structure prediction using *JPred*4 (Drozdetskiy *et al.*, 2015 ▸) of full-length Rco_pLS20_. The rectangles indicate the HTH domain at the N-terminus as annotated in UniProt entry E9RIY8 and the RcoTet_pLS20_ domain corresponding to the crystallized fragment. The amino-acid letters are colored according to secondary structure in the crystal structure (green for β-strand and red for α-helix). (*b*) Cartoon representation of residues Val125–Tyr155 of the RcoTet_pLS20_ dimer, colored by chain. All side chains are shown as sticks; the numbers of selected residues of the green monomer are indicated. A monomer of the p53 structure (PDB entry 1aie; Mittl *et al.*, 1998 ▸), superposed on the green monomer of Rco_pLS20_, is shown in transparent gray. (*c*) Side view of the tetrameric structure, showing the two charged layers formed by the Arg141 and Glu145 residues from the four monomers and the way each of these clusters is capped by a pair of Leu148 residues. (*d*) Close-up view of a single Arg141/Glu145 layer and Leu148 cap. The hydrogen bonds between the residues of the charged cluster are indicated by yellow dotted lines. (*e*) Electrostatic potential (ESP) on the surface of the tetrameric structure of RcoTet_pLS20_ in two orientations. The orientation shown in the left panel is similar to that shown in Fig. 1 ▸(*b*). In the right panel, RcoTet_pLS20_ is rotated by 90° around a vertical axis, showing the ESP of the view on the exposed face of the β-strands. (*f*) Superposed SEC elution profiles of RcoTet_pLS20_ at different pH values.

Crystallization trials on apo full-length (FL) Rco_pLS20_ were not successful, but cocrystallization of FL Rco_pLS20_ with various DNA sequences did result in the formation of crystals. However, structure determination revealed that the crystals contained only part of Rco_pLS20_, corresponding to residues Val125–Lys159 of the FL protein, and no DNA molecules (Fig. 1 ▸). Since the missing sequence of the protein cannot fit into the asymmetric unit, it is likely that the protein degraded during the course of the crystallization experiment, which often occurs in multidomain proteins. Indeed, size-exclusion chromatography (SEC) analysis of the protein confirmed that degradation occurred over time (Supplementary Fig. S1), leading to the appearance of fragments with a molecular weight compatible with the crystallized fragment. We show below that the crystallized fragment indeed corresponds to one of the domains of Rco_pLS20_, which we will refer to as RcoTet_pLS20_. The asymmetric unit contains four monomers forming one crystallographically independent tetramer.

Each monomer consists of an elongated sequence comprising residues Val125–Thr132, which includes a four-amino-acid β-strand formed by residues Arg127–Asp130. This strand is followed by a sharp turn facilitated by the glycine residue Gly133, which is followed by an α-helix comprising residues Glu136–Lys157 (Fig. 1 ▸ *a*). The RcoTet_pLS20_ tetramer consists of a dimer of primary dimers. The primary homodimer is formed by the arrangement of two β-strands from two monomers in an antiparallel fashion and by concomitant antiparallel packing of the helices against one face of the two β-strands (Fig. 1 ▸ *c*). The α-helices interact with one of the faces of the β-strands through hydrophobic interactions involving Val131, Phe129, Leu134, Ile139, Val142, Ile146 and Leu149 (Fig. 1 ▸ *b*). At the center of the exposed helical face, a cluster of charged residues is formed by Arg141 and Glu145. The hydrophobic Leu148 residues are located at both extremes of this cluster along the α-helix (Figs. 1 ▸ *c* and 1 ▸ *d*), which connects to the hydrophobic cluster through Leu149. The tetramer is formed through interactions of the Arg141, Glu145 and Leu148 residues, which we will refer to as the REL motif, from the four monomers (Figs. 1 ▸ *c* and 1 ▸ *d*). The carboxylic acid groups of the Glu145 residues interact through hydrogen bonds (Fig. 1 ▸ *d*).

The overall shape of the tetrameric structure is reminiscent of an octagon (see Fig. 1 ▸ *e*, left panel). The C-terminal ends of two helices form two pairs of opposed vertices of the octagon. The remaining four vertices are formed by the N-termini of the β-strands. Thus, two pairs of N-terminal DBDs are located on opposing sides of the RcoTet_pLS20_ domain. The lateral edges of the octagon formed by the β-strands are hydrophobic (Fig. 1 ▸ *e*), which is expected to contribute to interactions with the DBDs based on analogy with other structures (see below). The planar faces of the octagonal box are formed by α-helices (Fig. 1 ▸ *e*).

### 3.2. pH-dependent oligomerization behavior of Rco_pLS20_

Rco_pLS20_ tetramerization seems to mainly be driven by the charged interactions of the REL motif (Figs. 1 ▸ *c* and 1 ▸ *d*). This triggered us to study the oligomerization behavior of full-length Rco_pLS20_ at pH 5, pH 8 and pH 10, respectively. We found that Rco_pLS20_ tends to form higher order oligomers under alkaline conditions (pH 10; Fig. 1 ▸ *f*). Under acidic conditions, *i.e.* pH 5, disruption of the tetramer is observed (Fig. 1 ▸ *f*). It is likely that protonation-induced neutralization of the carboxylates of the central Glu145 occurs at pH 5. This causes disruption of the counter-charge stabilized hydrogen-bonding network, shown in Fig. 1 ▸(*d*), between the agglomerated Glu145 and Arg141 residues at the tetramerization interface.

The MW of the species that form at pH 5, pH 8 and pH 10 were estimated by calibration of the SEC column elution based on the individual elution of a set of proteins of distinct MW under equivalent conditions (Fig. 1 ▸ *f*). The MW estimates are 56.99 kDa at pH 5, 85.43 kDa at pH 8 and 151.57 kDa at pH 10 (Supplementary Table S1). Given that the MW of FL Rco_pLS20_ is 20.32 kDa, the elution peaks therefore correspond to two to three FL protein molecules at pH 5, four protein molecules at pH 8 and eight protein molecules at pH 10 (Supplementary Table S1). It is unlikely that Rco_pLS20_ can form a trimer at pH 5; it is far more likely that protonation of the Glu145 residues at pH 5 disrupts the tetramerization interface, resulting in dimers. The slight deviation in the elution pattern from a dimer at pH 5 may be a result of an altered surface charge under acidic conditions, which may affect interactions with the agarose–dextran matrix of the chromatography column, or a mixture of dimers and tetramers that results in an average migration of the peak. These data show that the protein prefers forming octamers at pH 10 even at low concentrations, which can be isolated by SEC. No direct evidence for hexamers, heptamers or complexes larger than octamers have been observed by SEC under the conditions tested.

### 3.3. Comparison of the TetD_loop_ folds of structural homologs

A search of the PDB (Berman *et al.*, 2000 ▸) for structural homologs of RcoTet_pLS20_ using *PDBeFold* (Krissinel & Henrick, 2005 ▸) resulted in several significant hits, which all corresponded to the human oncogene p53 and its analogs (Supplementary Table S2). The basic fold of all these structures consists of a pair of short β-strands followed by an α-helix connected by a kinked loop (Figs. 1 ▸ *a* and 2 ▸ and Supplementary Fig. S2). Hydrophobic residues line the internal surfaces of the β-strands and the α-helix, thereby forming the hydrophobic core of the structure. Both the β-strands and the α-helix have similar lengths, and structural differences are mainly found in the angle between the β-strands and the α-helix, which appears to be conditioned by the residues forming the kinked loop. In the structures identified and analyzed here this angle is smaller than 27° when a glycine is present in the loop, whereas it ranges from 34° to 62° when this glycine is lacking, as is the case for *Drosophila melanogaster* p53 (Dmp53) and CEP-1 (Supplementary Fig. S2). Due to the variation in the angle, the monomers of the structural homologs generally do not superpose, which complicates the detection of structural similarity. Strikingly, the structure of Rep_SPC32H_ (Kim *et al.*, 2016 ▸), a repressor encoded by *Salmonella* phage SPC32H, reveals a tetramerization domain that is structurally similar to RcoTet_pLS20_ and p53Tet. This domain is also called CAD and, apart from inducing tetramers in Rep_SPC32H_, it is also responsible for its interaction with the antirepressor Ant (Kim *et al.*, 2016 ▸). We propose that it is a TetD_loop_ and will refer to it as such here. This structure did not appear in the *PDBeFold* search using RcoTet_pLS20_ as a query as described above. Instead, Rep_SPC32H_ was identified as an Rco_pLS20_ analog based on the following shared features. Firstly, the architecture of the two full-length proteins is similar and contains a DBD at the N-terminus and a TetD_loop_ at the C-terminus. Rep_SPC32H_ additionally contains a dimerization domain (MDD) situated between the DBD and the TetD_loop_ (Kim *et al.*, 2016 ▸). Secondly, the DBDs are homologous in sequence. Thirdly, Rep_SPC32H_ is a transcriptional repressor and, together with its antirepressor protein Ant, exhibits a similar regulatory mechanism to the Rap_pLS20_–Rco_pLS20_ pair. Thus, Rep_SPC32H_ prevents entry into the lytic cycle of the phage by tight repression of the genes essential for the lytic cell cycle. However, the structural mechanism of gene repression of this protein has not been extensively characterized.

### 3.4. Relative spatial position of the TetD_loop_ and additional domains

**Figure 2:**
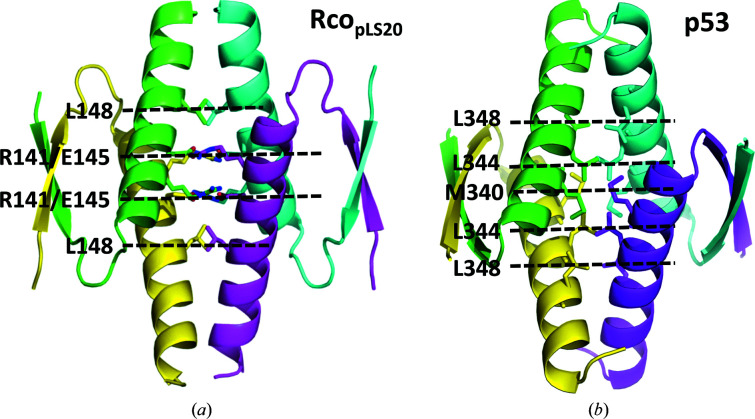
Structure of tetramer-forming domains of Rco_pLS20_ and human p53. (*a*) Cartoon representation of the structure of the tetramerization domain of Rco_pLS20_ (this work; PDB entry 8bny). (*b*) Cartoon representation of the structure of the tetramerization domain of p53 (PDB entry 1aie). The hydrophobic residues forming the interface are indicated.

The angle between the β-strands and the α-helix in the monomer ultimately determines the respective orientations of the dimers in the quaternary structure of the tetramer. This further complicates the detection of structural similarity between structural homologs. The interface of the tetramer is all hydrophobic for p53 (Fig. 2 ▸ *b*) and all of its homologs, except for Dmp53, which has a charged core similar to RcoTet_pLS20_, consisting of interacting glutamate residues lined with arginines in a two-layered configuration, as shown for RcoTet_pLS20_ in Figs. 1 ▸ and 2 ▸(*a*). Interestingly, the β-strands and α-helix are duplicated in the sequence of Dmp53, and the tetramer interface is therefore formed by eight helices, *i.e.* two helices from each of the four monomers. The exact nature of the amino acids involved in the hydrophobic tetramerization interfaces can vary, which is exemplified by Rep_SPC32H_ and p53: the former uses phenylalanines Phe187 and Phe190 of the four monomers to form this interface, whereas in p53 a cluster of leucine and methionine residues is found.

**Figure 3:**
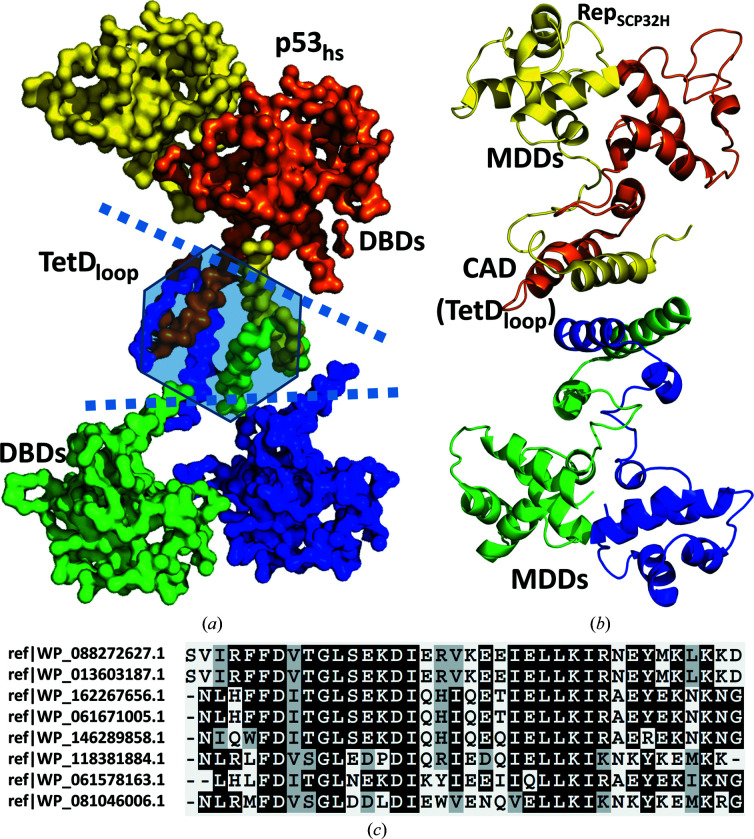
TetD_loop_ structures identified to be analogs of RcoTet_pLS20_. (*a*) C^α^ trace of the tetrameric electron microscopy structure (PDB entry 5xzc; Ghosh *et al.*, 2019 ▸) of full-length human p53 complexed with p300 (not included). The four monomers are colored differently. The DBD and tetramerization domains are labeled and the boundaries between these domains are indicated by dotted lines. (*b*) Cartoon representation of one of the tetramers from the structure of the phage repressor Rep_SPC32H_ (PDB entry 5d4z). (*c*) Multiple sequence alignment of the *B. subtilis* sequences retrieved by a *BlastP* search of the RcoTet_pLS20_ domain.

The quaternary structures of the TetD_loop_ of p53 and Rco_pLS20_ are similar (Fig. 2 ▸), despite the difference in the relative orientations of the dimers across the tetramerization interface. The configuration of the interaction between primary dimers causes the N-termini of the TetD_loop_ to point in opposite directions. This is confirmed by the FL structure of a complex of p53 and p300 determined by electron microscopy (Ghosh *et al.*, 2019 ▸), which shows a planar overall structure with the DBDs interacting pairwise; each pair extends away from the central p53Tet domain in opposite directions (Fig. 3 ▸ *a*). The placement of the MDDs in the structure of Rep_SPC32H_ is similar (Fig. 3 ▸ *b*), where two pairs of adjacent MDDs are separated by ∼100 Å, as measured between the far ends of these domains. The highest ranked *AlphaFold*2 model of a tetramer of full-length Rco (Supplementary Fig. S3*a* ) is consistent with the arrangement of the RHH domains of p53 and Rep_SPC32H_ shown in Figs. 3 ▸(*a*) and 3 ▸(*b*), respectively. It is interesting to note that the *AlphaFold*2 model of the tetramer of the full-length protein (Supplementary Fig. S3*b* ) shows that the tetramerization domain is well predicted for this oligomerization state.

### 3.5. Structurally similar proteins show low sequential homology

The vertebrate p53 homologs generally share low sequence homology in regions other than their DNA-binding domains (Ou *et al.*, 2007 ▸; Lu & Abrams, 2006 ▸). In line with this, RcoTet_pLS20_ and Rep_SPC32H_ also share low homology with these proteins. For example, the residues conferring interactions between the β-strands and the α-helix in the core of the dimer are hydrophobic, but the identities of these residues are not conserved between the different TetD_loop_ domains. Similarly, even within vertebrates the tetramerization interface has diverged among different proteins, leading to the occurrence of hydrophobic and/or charged tetramerization interfaces as described above. It is therefore not surprising that sequential homology is low in these structurally similar proteins.

Despite low sequence conservation, we used the RcoTet_pLS20_ domain (Rco_pLS20_ residues Ser124–Asp161) as a query in *BlastP* searches. As expected from the low sequence homology, none of the structural homologs described above were identified in the *BlastP* search. Instead, nine nonredundant proteins with *E*-values ranging between 3 × 10^−6^ and 2 × 10^−3^ were identified, which are all encoded by bacteria belonging to the phylum *Firmicutes* (Fig. 3 ▸ *c*). Thus, the TetD_loop_ is found in G+ proteins related to *B. subtilis*.

## 4. Discussion

Rco_pLS20_ is a transcriptional regulator that exerts its function through the formation of a DNA loop by binding to two regions that are separated by about 75 bp (Ramachandran *et al.*, 2014 ▸). The N-terminal HTH motif identified in the N-terminal region is likely to be involved in recognition of the rcoREs, but the structural basis of looping was not well understood up to this point. Our present results show that the master regulator Rco_pLS20_, which is crucial in the transcriptional regulation of the conjugation operons of plasmid pLS20, contains a tetramerization domain that is likely to be involved in formation of the DNA loop, and that this domain has high structural homology to the tetramerization domain present in the p53 family of tumor repressors. The structure reveals that RcoTet_pLS20_ forms tetramers like the p53 tumor suppressor protein family and shows how the different DNA-recognition elements are bridged. Identification of the tetramerization domain based on sequence was hampered by its low sequence homology with similar domains of known function.

Rco_pLS20_ is one of many examples illustrating the intimate link between protein tetramerization and DNA looping or long-range DNA cross-linking observed in both prokaryotic and eukaryotic systems. In prokaryotes, regulators have been extensively studied, for example *gal* and *lac* (Cournac & Plumbridge, 2013 ▸; Matthews, 1992 ▸), whereas in eukaryotes the p53 analogs (Kearns *et al.*, 2016 ▸; Stenger *et al.*, 1994 ▸) are well known examples. To date, however, no structural homology has been reported between the structures of tetrameric, DNA-loop inducing proteins from eukaryotes and non-eukaryotes. Remarkably, we found that the TetD_loop_ domain observed in Rco_pLS20_ and p53 is also present in the structure of *Salmonella* phage SPC32H (PDB entry 5d4z). However, structural homology of this domain to existing structures was not detected in this study (Kim *et al.*, 2016 ▸), although a structural comparison with PDB structures using *DALI* was performed. It is likely that variations in the sequence and in the relative orientation of the β-strands and α-helix hampered detection. In fact, the Rep_SPC32H_ linker consists of a short α-helix which is not present in any of the other structures. This connecting α-helix allows the β-strands and the second α-helix to adopt a nearly parallel configuration, without the need for a glycine residue. Furthermore, there is no sequence homology between the dimer and tetramer interface of the TetD_loop_ of Rep_SPC32H_, and all the proteins analyzed above.

Despite the low sequence homology, it was possible to discern motifs for the proteins that contain a charged tetramerization interface. In our analysis, a positively charged residue (Arg141 in RcoTet_pLS20_) should be followed approximately a full turn later in the α-helix (*i.e.* three or four positions downstream) by a glutamate (Glu145 in RcoTet_pLS20_). In addition, we find a hydrophobic residue at a second full turn (+7) from the positively charged residue (Leu148 in RcoTet_pLS20_). We named this distinctive motif the ‘REL motif’. Furthermore, a glycine residue is often found in the sharp turn between the α-helix and β-strands and may be of importance, as it is highly conserved in metazoan homologs of human p53 and in hits found through sequence-homology searches. From our comparison, it seems that a glycine is a prerequisite for a small angle between the β-strands and α-helix, as a lack of this residue leads to differences in the orientation of the helices and β-strands of the respective monomers. It should be noted that variations on this motif exist, as the Rep_SPC32H_ structure contains a short additional α-helix perpendicular to the preceding β-strand and the trailing α-helix (Fig. 3 ▸ *b*). Variability is also observed for the tetramerization interface joining two dimers, which has evolved to either a completely hydrophobic interface or an interface stabilized by complementary charges.

Rco_pLS20_ has the capacity to form higher order complexes as shown by the results described here and by SAXS analysis of FL Rco_pLS20_ at neutral pH, where it was shown to occur in a concentration-dependent manner (Crespo *et al.*, 2020 ▸). The pH dependence of octamerization determined here suggests that this interface is also charged, like that of the tetramer. Analysis of the distribution of charges and hydrophobic patches across the surface of RcoTet_pLS20_ suggests that the interaction is likely to be mediated by the charged face of the octagonal form of the tetramer, given that the edges are hydrophobic. Remarkably, inspection of the interactions between symmetry-related molecules of the crystal structure of Rep_SPC32H_ reveals interactions between the TetD_loop_ of adjacent tetramers, resulting in octamers that are in accordance with the model of octamerization through the TetD_loop_, as proposed for Rco_pLS20_. The model that emerges from the DNA binding of the tetramer is that of cooperative binding of recognition sites across the DNA loop. The initial RcoTet_pLS20_–DNA complex would be formed stochastically, perhaps with the aid of helper proteins that shape the DNA at the turn and through the intrinsic propensity of the DNA to bend at the loop region (Ramachandran *et al.*, 2014 ▸). Binding of several tetramers of the repressor across the loop stabilizes the loop structure.

The striking structural similarity between the prokaryotic and eukaryotic tetramerization domains exemplified by RcoTet_pLS20_ and p53, respectively, raises the question whether they evolved independently or whether they are derived from a common ancestor. This question cannot be answered straightforwardly, as sequence homology in these short sequences is difficult to detect. However, the conservation of the REL motif in some interfaces, coupled with the structural similarity of the core strand–kink–helix, suggests common ancestry. For example, RcoTet_pLS20_ and Dmp53 are structurally homologous and both have a charged tetramerization interface, albeit that duplication of the secondary-structure elements occurs in Dmp53 as described above. In addition, a well established cofactor of p53, named Strap, consists of a TPR motif (Adams *et al.*, 2012 ▸) and interacts with p53 (Jung *et al.*, 2007 ▸). Thus, Strap could be a functional homolog of Rap_pL20_.

The combination of a shared core structure and tetramerization interface and cofactors of similar structure suggests that convergent evolution is unlikely and strongly favors the hypothesis that all proteins incorporating a TetD_loop_ have diverged from a common ancestor. The presence of the TetD_loop_ motif across the gene pools of all kingdoms of life also supports divergent evolution, as it is unlikely to have occurred through multiple convergent events. We propose therefore that all members of this family stem from a common ancestor that included DBD and TetD_loop_ domains. This would imply that the TetD_loop_ fold predates the occurrence of multicellularity some 3–3.5 billion years ago (Grosberg & Strathmann, 2007 ▸).

The work described here suggests a common regulatory mechanism that exists in all kingdoms of life and that controls gene transcription, which is mediated by a tetramerization domain that we call TetD_loop_. The detection of this domain in sequences and structures is complicated due to low sequence conservation and to structural variation of the constituting elements. We describe the motifs that we have observed in the different structures. However, improved bioinformatics tools that can increase the predictive power for identifying small domains such as TetD_loop_ would help in the detection of these domains in proteins that share similar mechanisms conferred by this domain.

## Supplementary Material

PDB reference: tetramerization domain of Rco, 8bny

Supplementary Figures and Tables. DOI: 10.1107/S2059798323001298/jb5053sup1.pdf

## Figures and Tables

**Table 1:** Summary of the data-processing and refinement statistics for crystallo­graphic analysis of the RcoTet_pLS20_ structure (PDB entry 8bny) Values in parentheses are for the outer resolution shell.

Data collection	
Beamline	XALOC, ALBA
Wavelength (Å)	0.9793
Space group	*P*2_1_2_1_2_1_
*a*, *b*, *c* (Å)	35.33, 36.18, 109.53
Resolution range (Å)	34.36–1.409 (1.433–1.409)
No. of reflections	
Total	96338 (5832)
Unique	18842 (943)
Ellipsoidal completeness (%)	91.6 (51.9)
〈*I*/σ(*I*)〉	14.5 (1.7)
Average multiplicity	5.1 (6.2)
*R*_merge_ † (%)	3.9 (71.4)
*R*_meas_ ‡ (%)	4.4 (78.0)
CC_1/2_ (%)	99.9 (51.2)
Structure refinement	
*R*_cryst_ §/*R*_free_ ¶ (%)	23.32/24.91
R.m.s.d. from target values	
Bond lengths (Å)	0.0058
Bond angle distances (Å)	0.724
*MolProbity* scores	
Clashscore (‰)	2.0
Poor rotamers (%)	2.10
Ramachandran outliers (%)	0.00
Ramachandran favored (%)	97.0
Overall score (%)	1.41
Isotropic *B*-factor analysis	
Average model *B* factors (Å^2^)	38.49
*B* factor from Wilson plot (Å^2^)	35.0
No. of non-H atoms	1263
No. of solvent molecules	62

† *R*_merge_ = 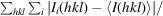 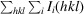 .

‡ *R*_meas_ = 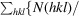 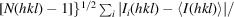 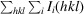 .

§ *R*_cryst_ = 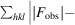 
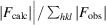
 .

¶ *R*_free_ = 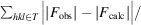 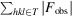 , where *T* represents a test set comprising ∼5% of all reflections that were excluded during refinement.
