# Supplementary material for: A tetramerization domain in prokaryotic and eukaryotic transcription regulators homologous to p53
Source: Acta Crystallogr D Struct Biol. 2023 Mar 1;79(Pt 3):259–67. doi: 10.1107/S2059798323001298 (PMC9986798; doi:10.1107/S2059798323001298)
Supplement: Supplementary file 1 [file d-79-00259-sup1.pdf]

Acta Cryst D

STRUCTURAL BIOLOGY

Volume 79 (2023)

Supporting information for article:

A tetramerization domain in prokaryotic and eukaryotic transcription regulators homologous to p53

Nerea Bernardo, Isidro Crespo, Anna Cuppari, Wilfried J. J. Meijer and D. Roeland Boer

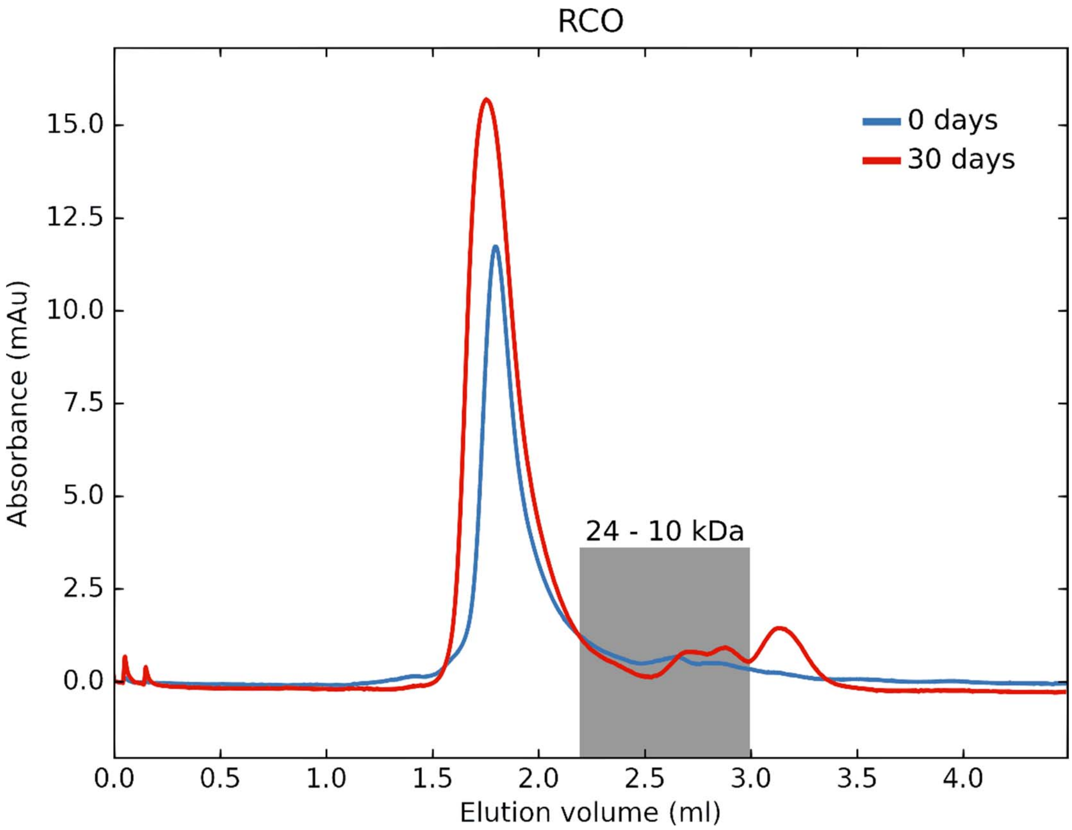

Figure S1 Analytical size exclusion chromatography (SEC) of RCO at different stages of degradation. The chromatogram for fresh Rco is shown in blue, and the chromatogram of a 30 days old Rco is shown in red. The shaded gray area covers the range of molecular weights from 24 kDa (2.168 ml) to 10 kDa (3.0 ml, corresponding to the total column volume according to the manufacturer). The monomeric crystallized fragment has a molecular weight of 4.3 kDa, the tetramer therefore corresponds to a molecular weight of 12.2 kDa.

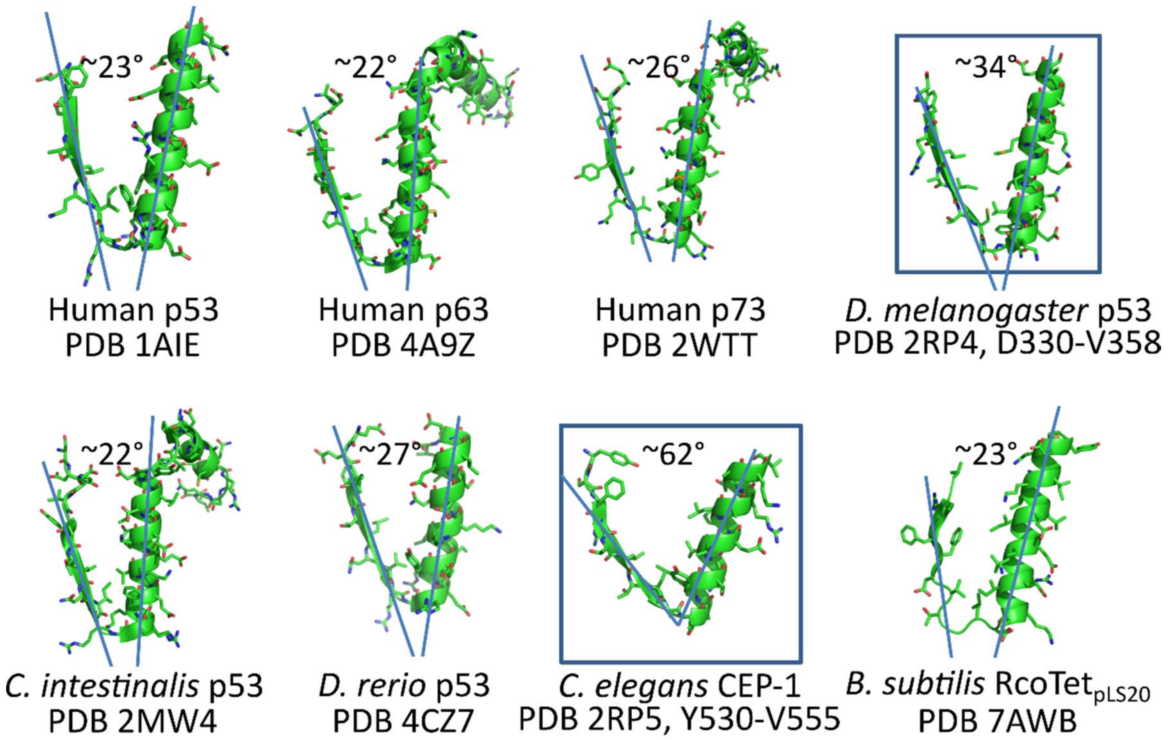

Figure S2 Cartoon and stick representation of the core strand-loop-helix motif of the RcoTet$_{pLS20}$-like structures. The angle between the helix and strand is shown. CEP-1 and Dmp53 are proteins that do not have a glycine in the loop region, and are highlighted by a square box.

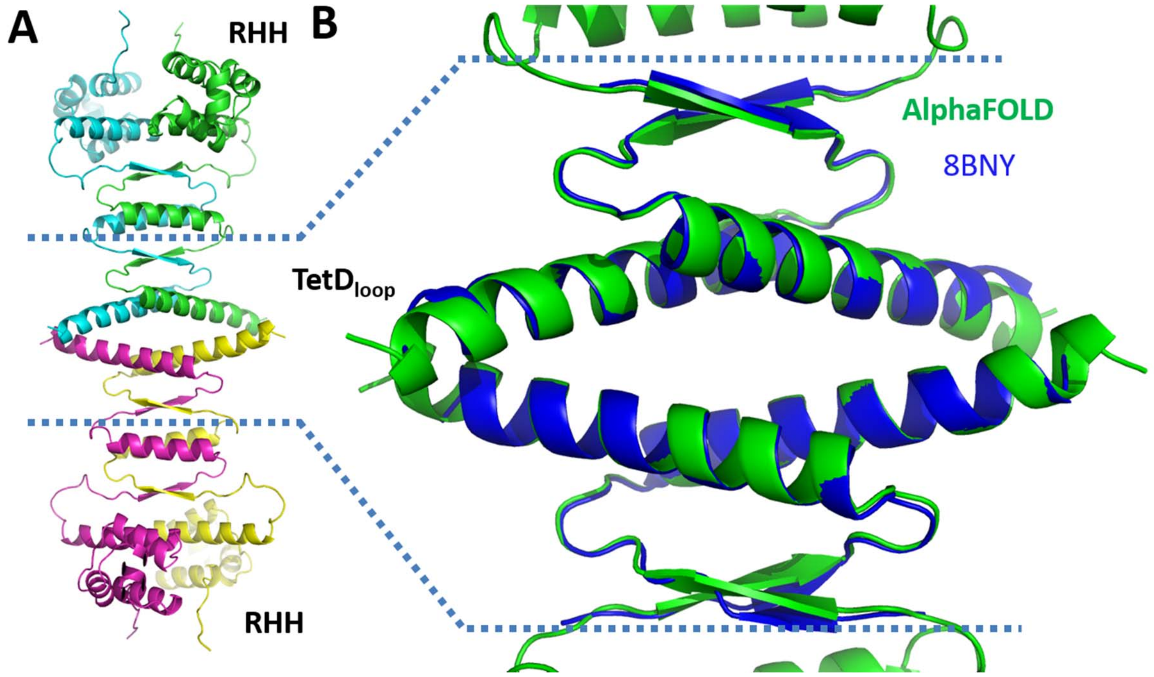

Figure S3 AlphaFold2 model of the full-length Rco dimer. A) Cartoon representation of the tetrameric model of the full length Rco_{pLS20} as calculated by AlphaFold2, colored by chain. B) Closeup of the TetDloop of the AlphaFold2 model (green), superposed on the X-ray structure presented herein (PDB code 8BNY, blue).

Table S1 Summary of the results obtained by SEC for Rco_{pLS20} at different pHs. The table shows the V_{el} of Rco_{pLS20} at pH5, pH8 and pH10, together with an estimation of MW and predicted oligomerization state. The theoretical MW of Rco_{pLS20} is 20.32 kDa.

pH	V_{el} (ml)	Estimated M_W based on V_{el} (kDa)	Estimated oligomerization state	Inferred oligomerization state in solution
5	1.97	56.99	2.8	Dimer
8	1.80	85.43	4.2	Tetramer
10	1.66	151.57	7.5	Octamer

Table S2 Hits retrieved by the eFold search using the $RcoTet_{pLS20}$ structure as subject and applying the standard cutoff of 70 % sse.

##	RMSD	Nalign	N	%seq	Query			Protein	Organism
					%sse	PDB	Nnes		
1	1.09-2.88	22-29	1	8-23	100	2wqj	26-32	P73Tet	*H. sapiens*
2	1.32-2.67	23-28	1	4-15	100	3zy0	26-29	P63Tet	*H. sapiens*
3	1.38-2.92	22-26	1	12-14	100	4cz7	30-31	P53Tet	*D. rerio*
4	1.54-1.70	29-30	1-2	13-14	100	1saf	42	P53Tet	*H. sapiens*
5	1.61-2.22	22-24	1	14-17	100	4cz6	26-33	P53Tet	*D. rerio*
6	1.63-1.90	29-30	1	17	100	2j10	31	P53Tet	*H. sapiens*
7	1.64-1.90	29-30	1	13-14	100	2j0z	31	P53Tet	*H. sapiens*
8	1.69	29	1	14	100	1sal	42	P53Tet	*H. sapiens*
9	1.69-1.85	29	1	14	100	2j11	31	P53Tet	*H. sapiens*
10	1.69	29	1	14	100	1sak	42	P53Tet	*H. sapiens*
11	1.70-1.71	29	1	14	100	1sae	42	P53Tet	*H. sapiens*
12	1.72	29	1	14	100	3sak	42	P53Tet	*H. sapiens*
13	1.73-1.8	29-30	1	13-14	100	1olh	42	P53Tet	*H. sapiens*
14	1.82-2.70	19-29	1	14-21	100	1olg	42	P53Tet	*H. sapiens*
15	1.9	23	1	17	100	2wtt	29	P73Tet	*H. sapiens*
16	2.18-2.95	24-26	1	12-13	100	4cz5	28-32	P53Tet	*D. rerio*
17	2.25	29	1	14	100	1hs5	34	P53Tet	*H. sapiens*
18	2.85	27	1	4	100	1a1u	29	P53Dim	*H. sapiens*
